# Long-Term Destiny of Corneal Endothelial Cells in Anterior Chamber Intraocular Lens-Implanted Eyes

**DOI:** 10.1155/2020/5967509

**Published:** 2020-12-24

**Authors:** Yating Tang, Jie Xu, Jiahui Chen, Yi Lu

**Affiliations:** ^1^Department of Ophthalmology and Eye Research Institute, Eye and ENT Hospital of Fudan University, 83 Fenyang Road, Xuhui District, Shanghai 200031, China; ^2^NHC Key Laboratory of Myopia (Fudan University), Key Laboratory of Myopia, Chinese Academy of Medical Science and Shanghai Key Laboratory of Visual Impairment and Restoration, Shanghai 200031, China

## Abstract

**Purpose:**

To investigate the long-term changes of corneal endothelial cells (EC) in anterior chamber intraocular lens- (AC-IOL-) implanted eyes.

**Methods:**

Retrospective study. We included 37 eyes (25 patients) that received AC-IOL implantation previously in the Eye and ENT Hospital of Fudan University between 1995 and 2016. Follow-up outcomes included the best-corrected visual acuity (BCVA), endothelial cell density, hexagonality, coefficient of variance, and central corneal thickness.

**Results:**

In total, 23 eyes (62.16%) with phakic and 14 eyes (37.84%) with aphakic AC-IOLs were included. Among these, 3 eyes (8.11%) were angle-supported AC-IOLs and 34 eyes (91.89%) were Artisan iris-fixated AC-IOLs. The mean age of patients was 41.40 ± 17.17 years, and the mean follow-up time was 12.12 ± 4.71 years in our study. At the follow-up time, corneal decompensation existed in 3 angle-supported AC-IOL eyes with a rate of 100% and 15 iris-fixated AC-IOL eyes with a rate of 44.12%. AC-IOL displacement occurred in 14 (41.18%) iris-fixated AC-IOL eyes. In the 19 iris-fixated AC-IOL eyes without corneal decompensation, significant changes also took place in corneal endothelial cells. The endothelial cell density decreased from 2843.26 ± 300.76 to 2015.58 ± 567.99 cells/mm^2^ (29.1% loss, *P* < 0.001) and hexagonality decreased from 51.21 ± 7.83 to 42.53 ± 9.17 (%) (16.9% loss, *P* < 0.001). The Kaplan–Meier survival curve also demonstrated the accumulated expectation rates of corneal endothelial cell decomposition for AC-IOLs with a median survival time of 12 years.

**Conclusion:**

We reported a significant chronic loss and long-term decompensation destiny of corneal endothelial cells in AC-IOL eyes. Semiannual or annual follow-up and evaluation of endothelial cells should be conducted in AC-IOL-implanted patients.

## 1. Introduction

Anterior chamber intraocular lenses (AC-IOLs) have been widely used in the past two decades in phakic myopic eyes and in aphakic eyes after a cataract extraction procedure. Given the relatively high efficiency and simplicity of the operation, Nuvita angle-supported and Artisan iris-fixated IOLs were the two most commonly and successfully used IOLs across all AC-IOLs in China. Though their effectiveness in correcting refractive errors has been soundly proven [1–3], their long-term safety and reliability are still an unresolved question, as most follow-up data of eyes implanted with angle-supported and iris-fixated IOLs were for less than five years [4–6].

The endothelial cell density is a key criterion for accessing the safety of AC-IOLs. The importance of endothelial cell density (ECD) loss for measuring the safety of the anterior chamber was emphasized by the American Academy of Ophthalmology (AAO) in early 2017 [7]. The AAO Task Force guidelines recommend that the percentage of eyes with a total ECD loss ≥25% after 3 years be used as an endpoint of investigation criterion. However, the AAO did not provide the long-term destiny prediction of AC-IOLs, considering that the data on long-term changes and safety (more than 10 years) of corneal endothelial cells were still urgently limited [3, 5, 8, 9]. Hence, in this study, we conducted a retrospective nonrandomized study of angle-supported and iris-fixated IOLs with a mean follow-up time of over 12 years. Our study aimed to provide some predictive data to foresee the long-term trend of corneal endothelial cells in AC-IOL-implanted eyes.

### 1.1. Patients and Methods

Patients who were implanted with angle-supported and iris-fixated AC-IOLs at the Eye and ENT Hospital of Fudan University, Shanghai, between 1995 and 2016 were identified through surgical records (*n* = 42, 57 eyes). From these patients, 13 could not be reached by telephone and 4 refused to be involved in the study; therefore, from the original group, 25 patients (37 eyes) had follow-up data available. This study adhered to the tenets of the Declaration of Helsinki. Approval was obtained from the Human Ethics Committee of the Eye and ENT Hospital of Fudan University, and all patients signed a statement of informed consent before all treatment and follow-up.

Inclusion criteria were patients older than 20 years at the implantation of Nuvita angle-supported AC-IOLs (B&L Nuvita MA20, Baush & Lomb, USA) and older than 6 years for Artisan iris-fixated AC-IOLs (Ophtec B. V., the Netherlands). The Nuvita angle-supported AC-IOL is a one-piece, polymethyl methacrylate (PMMA), rigid lens with a biconcave optical zone of 6 mm. The iris-fixated AC-IOL is a one-piece, PMMA, rigid lens with a convex-concave optic and an optical zone of 5 mm.

All the enrolled patients were angle-supported or iris-claw AC-IOL implanted, had an anterior depth (ACD) at the period of implantation of 3 mm or more, and had an endothelial cell density ≥2000 cells/mm^2^ in patients ≥25 years and ≥2500 cells/mm^2^ for younger patients. Exclusion criteria were previous intraocular surgery, glaucoma, eye trauma, uveitis, and corneal diseases that might have had influence on the endothelial cells before the AC-IOL implantation surgery. For aphakic eyes, patients underwent cataract extraction surgery, in addition to the inclusion and exclusion criteria above.

For each patient, we recorded the age at implantation, the age at explantation (if it continued), and age at corneal decompensation (if it occurred). We also recorded patient complications during the whole long-term follow-up (glaucoma, corneal decompensation, uveitis, cataract, and retinal detachment). Best-corrected visual acuity (BCVA), slit-lamp microscopy, intraocular pressure (IOP), and fundus examination after pupil dilation were performed at all follow-up visits. The corneal endothelial cells were measured using the Tomey specular microscope (EM-3000, Tomey, Tokyo, Japan).

The method of implantation of angle-supported and iris-fixated AC-IOLs was conducted under local peribulbar anesthesia. All eyes achieved miosis status preoperatively using pilocarpine. All eyes were treated with an identical surgical procedure for the two AC-IOLs. After the 5 mm corneoscleral incision was centered at 12 o'clock, the viscoelastic substance (Discovisc, Alcon, USA) was injected sufficiently into the anterior chamber to support the anterior chamber depth, to protect the endothelial cells, and to avoid lens-touching during implantation. The angle-supported and iris-fixated IOLs were implanted into the anterior chamber. For angle-supported IOLs, the IOL was inserted in the anterior chamber with the distal haptics directed to the angle. The IOL was gently rotated at the horizontal axis. A slight adjustment of haptics was conducted to avoid pupil ovalization. For Artisan IOLs, after being rotated at the horizontal axis, the claws were fixed to the midperipheral iris stroma. For all eyes, a slit iridotomy was performed at 12 o'clock to avoid pupillary block glaucoma. The viscoelastic substance was exchanged for balanced salt solution, and the incision was sutured with 3–5 interrupted 10-0 nylon sutures. After surgery, topical levofloxacin and prednisolone acetate were used 3 times daily for 3 to 4 weeks.

We analyzed the data using SPSS statistics version 24.0 (IBM/SPSS, Inc., Chicago, IL). Frequency (%) was used for the analysis of qualitative variables, and the mean and standard deviation (mean ± SD) were used for the analysis of quantitative variables. The comparisons between preoperative and last follow-up points were performed by paired *t*-test. Kaplan–Meier survival curves were used to compare corneal decompensation rates. Stepwise multivariate logistic regression procedures were conducted to evaluate the independent risk factors of corneal endothelium decompensation. The ORs and 95% confidence intervals are presented. Statistical significance was defined as *P* ≤ 0.05.

## 2. Results

**Figure 1:**
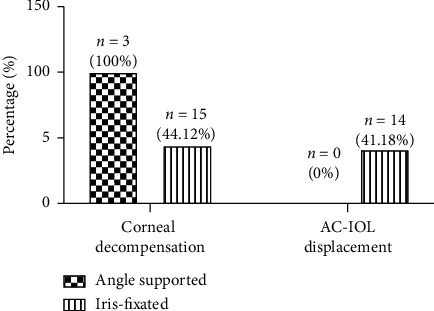
Long-term proportion of corneal endothelial decompensation and AC-IOL displacement in angle-supported and iris-fixated AC-IOLs. AC-IOLs: anterior chamber intraocular lenses.

At the last follow up, 25 patients (37 eyes) successfully completed the study. The characteristics of the study population are summarized in Table 1. The mean follow-up time was 12.12 ± 4.71 years. A total of 3 eyes (8.11%) were implanted with angle-supported IOLs, and 34 eyes (91.89%) were implanted with Artisan IOLs. At the follow-up time, corneal endothelial decompensation occurred in 18 eyes (48.65%) (Figure 1), with the mean corneal decompensation time of 10.17 ± 5.19 years. Corneal decompensation occurred in all 3 angle-supported eyes (100%) and 15 iris-fixated eyes (44.12%). AC-IOL displacement occurred in 14 (41.18%) iris-fixated AC-IOL eyes (Figure 1). In the 18 corneal endothelial decompensation eyes, all eyes (100%) received IOL explantation and 11 eyes (61.11%) received penetrating keratoplasty (PKP) or Descemet's stripping automated endothelial keratoplasty (DSEK) surgery. Among the 19 iris-fixated AC-IOL eyes without corneal decompensation, 16 eyes (84.21%) received IOL explantation surgery. In total, 34 eyes (91.89%) received AC-IOL explantation surgery because of cataracts, marked endothelial cell loss, or corneal decompensation. The mean AC-IOL explantation time was 9.37 ± 4.45 years.

**Figure 2:**
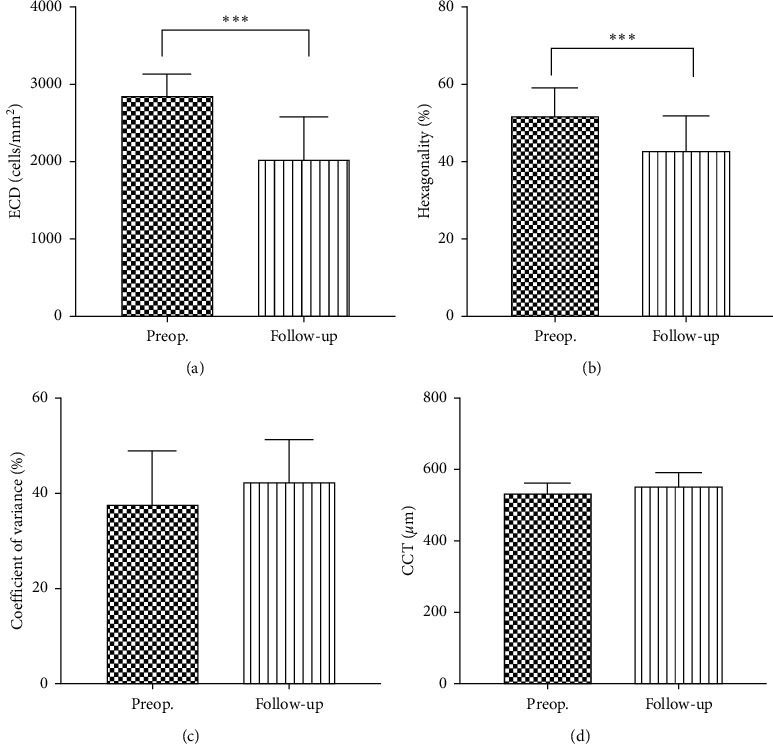
Long-term of ECD, hexagonality, coefficient of variation, and CCT changes in eyes without corneal endothelial cell decompensation. ECD: endothelial cell density; CCT: central corneal thickness.

For chronic endothelial cell changes in the 19 iris-fixated AC-IOL eyes without corneal decompensation, we also found a significant decrease of ECD and hexagonality (Figure 2(a) and 2(b)). The endothelial cell density decreased from 2843.26 ± 300.76 to 2015.58 ± 567.99 cells/mm^2^ (29.1% loss, *P* < 0.001) and hexagonality decreased from 51.21 ± 7.83 to 42.53 ± 9.17 (%) (16.9% loss, *P* < 0.001). We also found an increasing trend in the coefficient of variation and central corneal thickness; however, it was not significant (Figures 2(c) and 2(d)).

**Figure 3:**
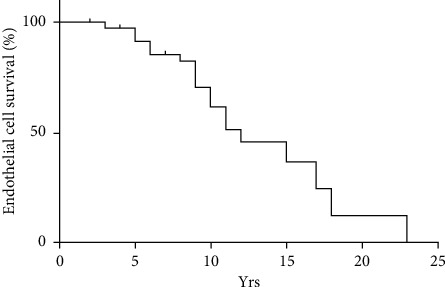
Kaplan–Meier survival curve showing the accumulated expectation rates of corneal decomposition for AC-IOLs. yrs: years.

We next used Kaplan–Meier survival curves to predict the corneal endothelial cell survival rates in AC-IOL-implanted eyes (Figure 3). The first onset of corneal decompensation was 3 years at follow-up, and the median survival time was 12 years. We next used a stepwise multivariate logistic regression model to study the independent risk factors of corneal endothelium decompensation. We included sex, age, preoperative ACD, the duration of time the AC-IOL was in the eye, IOL displacement, glaucoma, and uveitis. However, no significant independent risk factor was found in our model (all *P* > 0.05).

## 3. Discussion

Reporting the long-term changes of corneal endothelial cells on AC-IOLs is critical to provide evidence for establishing implantation guidelines and follow-up strategies. However, few studies have followed up patients for 10 years or more, and data for Chinese patients are even more rare. In the current study, we conducted a retrospective study of corneal endothelial cell changes with the mean follow-up time of 12.12 ± 4.71 years. We found that, at the follow-up time of 12 years, corneal endothelial cell decompensation had occurred in approximately half of the eyes. Furthermore, we also found a significant decrease in ECD (approximately, 30% loss) and hexagonality (16.9% loss) in eyes without corneal decompensation. We believe our patient data are valuable for understanding long-term outcomes and the safety of AC-IOLs in clinics.

Over the past years, most studies have focused on refractive and visual outcomes of AC-IOLs in myopic and pseudophakic eyes [10, 11]. In January 2017, the AAO Task Force published a special report with regard to the endothelial cell data for new phakic as well as pseudophakic IOLs. The guideline indicated that eyes with ≥25% ECD loss 3 years after implantation should be considered an endpoint for clinical investigation of Artisan IOL [7]. Another guideline made by the French Health Products and Safety Agency (AFSSAPS) suggested an ECD loss <1500 cells/mm^2^ or a total EC loss of 30% as a threshold for AC-IOL explantation [12, 13]. This group assumed that 1500 cells/mm^2^ is an important threshold to safely perform phakic IOL explantation and combined cataract surgery without compromising the long-term integrity of the corneal endothelium. However, both guidelines were based on short follow-up times, and most studies on ECD loss were short-term research [5, 14]. Given that most patients with implanted AC-IOLs were young people, and the 10 years or more of data on the corneal endothelium should also be considered in decision-making for the explantation of AC-IOLs. Jonker et al. [12] indicated that, for iris-fixated IOLs, the median end-point survival time (defined by AAO or AFSSAPS) was 15 years in implanted myopic eyes. Alio et al. [8] found that, for angle-supported IOLs, the median time to explantation was 12.3 years. In our study, we also found that the median survival time of the corneal endothelium was 12 years, which is mainly consistent with previous studies and much earlier than that of Jonker's study. In our study, AC-IOLs were explanted in 34 eyes (91.89%), with the mean explantation time of 9.37 ± 4.45 years. Though the explantation rate was much higher, there were still 18 eyes in which corneal endothelial decompensation occurred and another 2 eyes with ECD <1500 cells/mm^2^ in the noncorneal decompensation group. We also noticed that the earlier the AC-IOLs were explanted, the less ECD loss happened. Considering the annual linear decrease of ECD in AC-IOLs [12, 15, 16], we strongly recommend annual or semiannual examination using a specular microscope, and earlier explantation of AC-IOLs of less than 10 years would be helpful.

Several studies have found that a small ACD and a small edge distance were potential risk factors for ECD loss [15, 17, 18]. Jonker [12] and Shajari et al.[18] found that ACD <3.00 mm and small peripheral edge distance <1.0 mm were associated with significantly higher ECD loss in short- (4 and 5 years) and long-term (10 years) follow-up (HR 5.69 and HR 5.35, respectively, both *P* < 0.001). However, in our study, we did not found that small ACD was an independent risk factor for corneal decompensation. Two reasons might contribute to our result. First, we just included ACD ≥3 mm patients when implanting AC-IOLs in our clinical practice and excluded eye trauma and glaucoma patients. Second, most people in our study were much younger (mean age 41.40 ± 17.17 years old), and this age difference will result in deeper ACD to some extent. However, we believe that small ACD and edge distance will surely induce higher ECD loss and corneal decompensation because it accelerates the contact and inflammation between the AC-IOL and corneal endothelium, leading to ECD loss and corneal endothelium damage in the end.

Hexagonality proportion is another important indication in our study. Zheng et al. [19] have studied the hexagonal cells in the central region of the cornea by confocal microscopy and found the mean percentage to be 50 ± 11%, which is consistent with our preoperative data. It has been demonstrated that when endothelial cells are exposed to damage, the hexagonal endothelial cells lose their hexagonal shape and become irregular in shape and size, resulting in the inability to tolerate damage [20]. In our study, we also found a significant decrease in the hexagonal cell proportion over time. In the 18 eyes with corneal endothelium decompensation, the hexagonal cells were 0; in eyes without corneal endothelium decompensation, compared with the preoperative data, the hexagonal proportion decreased by 16.9%. We also noticed that, in the corneal endothelium decompensation group, the ECD could be relatively normal (even >2000 cells/mm^2^) but with irregular shape and 0 hexagonal cells. Thus, we speculated that hexagonality proportion decrease might take place much earlier than ECD loss in a stressed environment. Hexagonality cell decrease might be more objective and accurate for early prediction of ECD loss and corneal endothelium damage. Hexagonal cell change should also be noted in the guidelines and taken into consideration when we make explantation decisions.

The strengths of our study are that it included a relatively large and long-term follow-up with standard examination and detailed data for the eyes of Chinese patients. However, several limitations also exist. This is a retrospective study, and we did not measure the preoperative refraction. Furthermore, considering the fact that some patients did not complete regular annual follow-up, in some cases, we obtained the corneal decompensation onset time from the patients' memory and complaints, and thus, the corneal decompensation diagnosis might have been slightly delayed.

In conclusion, this study demonstrated that, after the implantation of AC-IOLs, the long-term changes of the corneal endothelium were not positive and were somewhat more disappointing than the short-term results. We strongly recommend regular annual or semiannual follow-up to be conducted in patients after AC-IOL implantation. International guidelines are urgently needed to set the threshold regarding ECD and hexagonal cell loss in the case of corneal decompensation in the long run.

## Figures and Tables

**Table 1:** Demographic data for the enrolled patients.

Characteristics	Angle-supported AC-IOL	Artisan IOL
Eye (participates)	3 (3)	34 (22)
Gender (male, %)	1 (33.33)	13 (59.1)
Age (year)	41.00 ± 7.55	41.45 ± 18.20
Mean ± SD (range)	(34–49)	(13–67)
Follow-up (year)	17.33 ± 5.51	11.41 ± 4.25
Mean ± SD (range)	(12–23)	(2–20)	
Right eye (%)	1 (33.33)	20 (58.82)
Phakic AC-IOL (%)	3 (100)	20 (58.82)
Aphakic AC-IOL (%)	0 (0)	14 (41.18)
Preop. AXL (mm)	27.13 ± 2.24	28.92 ± 3.99
Preop. ACD (mm)	3.52 ± 0.41	3.54 ± 0.37

AXL: axial length; ACD: anterior chamber depth.

## Data Availability

The data used to support the findings of this study are available from the corresponding author upon request.

## Supplementary Materials

Supplementary Materials Original data of the manuscript.
