# Supplementary material for: Long-Term Destiny of Corneal Endothelial Cells in Anterior Chamber Intraocular Lens-Implanted Eyes
Source: J Ophthalmol. 2020 Dec 24;2020:5967509. doi: 10.1155/2020/5967509 (PMC7783513; doi:10.1155/2020/5967509)
Supplement: Supplementary Materials — Original data of the manuscript. [file 5967509.f1.pdf]

编号	性别	age	随访时间mont	眼别	眼别	AL
1	1	64	8	R	1	23.87
2	2	42	15	L	2	34.53
3	2	42	15	R	1	35.67
5	1	51	12	L	2	22.19
4	1	51	12	R	1	22.34
6	1	52	11	R	1	25.39
7	1	61	8	R	1	22.86
8	1	22	9	R	1	26.63
9	1	40	20	L	2	25.7
10	2	42	12	R	1	32.06
11	2	41	13	L	2	34.48
12	2	41	13	R	1	34.57
13	1	27	11	L	2	32.56
14	1	27	11	R	1	33.32
15	1	67	9	R	1	26.49
16	2	49	12	R	1	
17	1	17	9	L	2	28.56
18	1	17	9	R	1	28.71
19	1	63	2	R	1	24.05
20	1	21	12	L	2	25.56
21	1	21	12	R	1	24.54
22	2	64	18	L	2	30.68
23	2	64	18	R	1	28.41
24	1	38	14	L	2	25.02
25	2	20	17	R	1	27.75
26	2	65	17	L	2	28.4
27	2	65	17	R	1	28.61
28	2	40	11	L	2	28.82
29	2	40	11	R	1	29.44
30	2	48	6	R	1	34.64
31	2	48	6	L	2	33.81
32	1	40	23	L	2	
33	1	14	9	L	2	29.56
34	1	14	9	R	1	28.85
35	2	13	8	R	1	32.21
36	2	13	8	L	2	32.86
37	2	34	17	L	2	

	ACD	是否取出手	植入-取出间	IOL眼内植入	失代偿时间	是否发生内
	3.49	1	3.5	3.5		2
	4.47	1	11	11	11	1
	4.61	1	11	11	11	1
	3.48	1	4	4	4	1
	3.21	2		12		2
	3.59	1	3	3	3	1
	3.5	1	6	6	6	1
	3.67	1	5	5	5	1
	3.4	1	18	18	18	1
	3.08	1	9	9	9	1
	3.51	1	12	12		2
	3.64	1	12	12		2
		1	10	10		2
		1	10	10		2
	3.45	1	8	8	8	1
3.18		1	11	11		2
	3.83	1	8	8		2
	3.61	1	8	8		2
	4	1	2	2		2
	3.32	1	9	9		2
	3.21	1	9	9	9	1
		1	10	10	10	1
		1	9	9	9	1
	3.52	1	6	6	6	1
	3.52	1	10	10	10	1
	3.75	1	15	15	15	1
	3.45	1	15	15		2
	3.13	1	9	9	9	1
	3.13	1	9	9		2
	3.36	1	4	4		2
	3.38	2		6		2
3.97		1	23	23	23	1
	3.12	1	7	7		2
	3	1	7	7		2
	3.86	2		8		2
	3.84	1	8	8		2
3.41		1	17	17	17	1

角膜移植	AC-IOL脱位	角膜水肿/混浊	术前青光眼	术前葡萄膜	术前角膜内皮	术前CV
	1	1	2	1	2524	47
1	1	1	2	2	N/A	
1	1	1	2	1	N/A	
2	1	1	2	2	N/A	
	1		2	2	2546	34
1	2	1	2	2	2586	缺
1	1	1	2	2	2624	32
2	2	1	2	2	N/A	
2	2	1	2	2	N/A	
1	2	1	2	2	N/A	
	2	2	2	2	2976	40
	2	2	2	2	2915	47
	2	2	1	2	2944	38
	2	2	1	2	2609	30
1	2	1	2	2	N/A	
	2		2	2	2512	40
	2	2	2	2	3133	50
	1	2	2	2	2900	50
	1	2	2	2	2500	34
	2	1	2	2	2562	32
2	1	1	2	2	N/A	
1	2	1	2	2	N/A	
1	1	1	2	2	N/A	
1	1	1	2	1	N/A	
1	2	1	2	2	3546	13
2	2	1	2	2	N/A	
	2	2	2	2	3196	36
1	1	1	2	2	2649	
	1	2	2	2	2581	35
	2	2	2	2	2494	36
	2	2	2	2	3194	35
2	2	1	2	2	2334(对侧眼)	31
	2	2	2	2	3437	14
	2	2	2	2	3019	10
	2	2	2	2	3204	39
	1	2	2	2	2776	61
2	2	1	2	2		

术前6A	术前CCT	随访视力BC	随访角膜内	随访CV	随访6A	随访CCT
57	560	0.8	2194	42	42	592
		0.2	2059	35	29	670
		0.1	1364	64	15	647
57	495	0.4	1575	33	47	487
缺	缺					
缺	564					
		0.4				
47	587	0.4	1492	35	43	514
44	559	0.7	1690	50	33	504
58	548	0.2	1817	30.6	50	512
64	548	0.3	1146	30.4	47	488
45	459	0.4	1523	61	33	511
45	490	0.7	1523	61	33	511
48	511	1	1746	47	33	627
54	526	0.15	2691	47	46	626
56	570	0.08	2180	47	39	610
28	574					
38	548	0.6	2799	46	35	556
52	531	0.8	1652	37	45	560
35	548	0.5	1503	39	25	550
64	520	0.6	2890	34	61	529
58	537					
50	511	0.8	2194	42	42	592
50	530	0.9	1965	47	45	573
57	530	0.3	3120	41	51	549
52	537	1	2596	37	58	563

随访时并发症	NCT
无	17
无	12
无	10
无	12.3
无	15
无	17
无	
无	
无	17
无	15
无	18
无	19
无	
无	
无	
无	21
无	23
无	17
无	18
	15
眼红，眼部[illegible]	11.6
